# The Thermal Gelation Behavior and Performance Evaluation of High Molecular Weight Nonionic Polyacrylamide and Polyethyleneimine Mixtures for In-Depth Water Control in Mature Oilfields

**DOI:** 10.3390/ma13184142

**Published:** 2020-09-17

**Authors:** Yi Qin, Ruiquan Liao, Shunshe Luo, Junliang Li

**Affiliations:** 1 Hubei Cooperative Innovation Center of Unconventional Oil and Gas, Yangtze University, Wuhan 430100, China; 201573006@yangtzeu.edu.cn; 2 College of Petroleum Engineering, Yangtze University, Wuhan 430100, China; 100619@yangtzeu.edu.cn (R.L.); lijunliang01@163.com (J.L.)

**Keywords:** nonionic polyacrylamide, gelation time, plugging capacity, water shutoff

## Abstract

A delayed crosslinked polymer gel was developed for in-depth water control in mature oilfields. The thermal gelation behavior of nonionic polyacrylamide (NPAM) and PEI was investigated, and sodium citrate (NaCit) was selected as a new retarder to prolong the gelation time. The gelation performance of NPAM/PEI gel system can be adjusted by varying NPAM or PEI concentration, and a quadratic model is developed by statistical analysis, which predicts the gelation time of NPAM/PEI gel system. The obtained model shows high significance and good reliability, as suggested by the F-ratio of 175.16 and high adjusted R-square value (0.9732). The addition of NaCit exhibits a good delayed gelation effect on the NPAM/PEI gel system, better than that of NaCl. The decrease of the initial pH value of the gelling solution leads to the weaker gel viscosity and longer gelation time due to the protonation of amine groups on the PEI chains. Increasing temperature results in higher gel viscosity but shorter gelation time. The gel system in the presence of NaCit exhibits good compatibility with injection and formation water. A dense three-dimensional structure was observed in matured NPAM/PEI/NaCit gel, and it could keep stable below 160 °C. The gel system could effectively reduce the permeability (>95%) and restricted the flow of water after matured in natural cores.

## 1. Introduction

Due to long-term water flooding for enhancing oil recovery, many high permeability channels are formed in the reservoir. It results in an increase in water productivity and the reduction of swept volume [1]. Moreover, excessive water production leads to many problems, such as pipeline and equipment corrosion, increased production cost, and environmental pollution [2]. Therefore, it is important to reduce the water-cut in mature reservoirs. Due to the adjustable gelation time and gel strength, compatibility with formation fluids, and extensive applicable temperature, crosslinked polymer gels have been used as one of the cost-effective technology for reducing water-cut and improving sweep efficiency in mature oilfield [3,4], especially the polyacrylamide-based crosslinked polymer gels [5].

The hydrolyzed polyacrylamide contains many amide groups (-CONH_2_) and carboxyl groups (-COO^−^) in its molecular chain. It could crosslink with different types of crosslinkers, such as Cr^3+^ [6,7], Al^3+^ [8], Zr^4+^ [9], phenolic resin [10,11], etc. However, multivalent metal ions and phenolic resin are toxic. Thanks to the eco-friendly and high nucleophilicity of polyethyleneimine (PEI), PEI crosslinked polymer gels have obtained a huge interest for water control treatment [12].

Previous studies have shown that different acrylamide-based polymers were developed to form stable crosslinked polymer gels with PEI. For instance, the copolymer (PAtBA) synthesized by acrylamide (AM) and t-butylacrylate (tBA) could form a rigid gel with PEI at high temperature and exhibited excellent plugging capacity in porous media [13]. Besides, the copolymer prepared with AM and 2-acrylamido-2-methylpropane sulfonic acid (AMPS) [14] and the terpolymer synthesized by AM, AMPS, and N-vinylpyrrolidone (NVP) [15] were reported to have potential application in high-temperature reservoirs (>100 °C). However, these polymers are expensive.

As an inexpensive alternative, polyacrylamide (PAM) was utilized to replace the copolymer and terpolymer to crosslink with PEI, and the gel system composed of PEI and various polyacrylamides were suitable for a wide range of temperature (23.3–130 °C) [16,17,18,19,20,21,22]. To ensure the gelling solutions permeate deep into the formation, inorganic salts, such as NaCl or NH_4_Cl, were added into the PAM/PEI gel system to delay the gelation time [16,17,21]. However, the retarding effect of NaCl was not satisfactory, while the addition of NH_4_Cl reduced the gel strength significantly. Jayakumar et al. pretreated PEI with AMPS or dextran sulfate (DS), respectively, and then cross-linked with partly hydrolyzed polyacrylamide (PHPA), which significantly extended the gelation time of PHPA/PEI gel system [23,24]. This approach is not conducive to large-scale application in oilfields due to the inconvenience of preparation and high cost.

Moreover, the structure property of PAM has a great influence on the thermal gelation of PAM/PEI gel systems. Jia et al. [19,20] reported that the gel formed by high molecular weight HPAM and PEI showed better thermal stability than that formed by low molecular weight HPAM at 40 and 65 °C at the same concentration, due to its more complex network skeleton. The larger is the molecular weight of the polymer, the stronger is the gel strength [22]. The dosage of polymer with high molecular weight was relatively lower than that of low molecular weight polymer to obtain the same gel strength. It is in favor of reducing the expenditure for water shutoff treatment.

However, the larger is the molecular weight, the shorter is the gelation time [22]. It is well known that the gelling solution prepared with high molecular weight PAM possesses high initial viscosity. This would make it difficult on the injection process for water shutoff treatment. Fortunately, gel systems prepared with nonionic polyacrylamide (NPAM) were studied. The low initial viscosity before crosslinking and good shearing resistance of NPAM-based gels showed the potential application for in-depth water control in mature reservoirs [25,26].

We notice that the investigations of NPAM-based gel were concentrated in the crosslinking of NPAM and phenolic resin. No systematic investigation was reported on the crosslinking of NPAM and PEI. In addition, based on the existence of ionic interaction between the carboxylate groups (-COO^−^) of polymer and the ammonium ions (NH_3_^+^) of PEI [27], organic acid salts may have the potential to be a retarder for prolonging the gelation time of PEI crosslinked gel. In this study, the thermal gelation behavior of the NPAM/PEI gel system was systematically investigated. Sodium citrate (NaCit) was selected as a new retarder to replace the traditional inorganic salts and delay the gelation time of NPAM/PEI system. The statistical analysis was conducted to develop the prediction mathematical model of gelation time, and the compatibility, microstructure, thermal stability, and plugging capacity of this gel system were evaluated for water control treatment.

## 2. Materials and Methods

### 2.1. Materials

The nonionic polyacrylamide (NPAM) was purchased from Guangzhou Shuirun Chemical Technology Co., Ltd. (Guangzhou, China), in which the molecular weight is 12 million Daltons (as provided by the manufacturer). A liquid form PEI with 50% purity was used as a crosslinker in this study, which provided by Guangzhou Mai Lichen CO., Ltd. (Guangzhou, China), and its molecular weight is 70,000. Additives including sodium chloride (NaCl), sodium citrate (NaCit), ammonium chloride (NH_4_Cl), and hydrochloric acid (HCl) were supplied by Guo Yao Chemical Co., Ltd. (Shanghai, China), which were AR grade. Fresh water, injected water, or formation water was used in these experiments. The injected and formation water was provided by Petrochina Xinjiang oilfield Co., Ltd (Karamay, China), and their ionic content is given in Table 1. Natural cores used in core flowing experiments were also provided by Xinjiang oilfield. No further purification treatment was performed for all chemical agents used in these experiments.

### 2.2. Methods

#### 2.2.1. Preparation of Gelling Solutions

All gelling solutions were prepared at room temperature. A certain concentration of NPAM was added into water and stirred until the homogeneous viscous solution was obtained. Then, the prepared polymer solution was aged for 12 h at room temperature. After that, a predetermined amount of PEI was mixed with the polymer solution with 10 min of stirring. Note that PEI was diluted as a 5% aqueous solution prior to use. The pH value of the gelling solution was adjusted by adding 1 mol/L hydrochloric acid. Note that the salt additives were preferentially soluble in water while preparing the samples.

#### 2.2.2. Determination of Gelation Time and Gel Viscosity of NPAM/PEI Gel System

**Figure 1:**
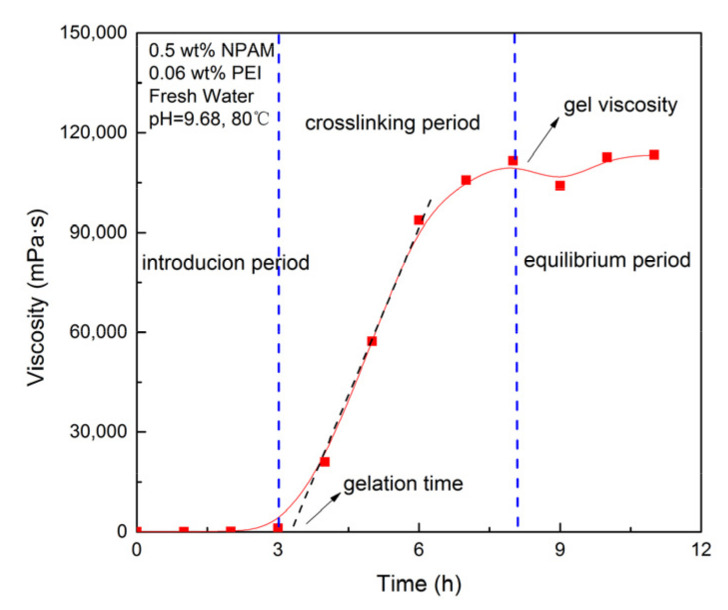
Crosslinking reaction process of the NPAM/PEI gel system.

Apparent viscosity measurements were conducted to quantitatively evaluate the gelation performance of NPAM/PEI gel system. The gelation process transformed from solution to gel was characterized by the variation of apparent viscosity correlated to reaction time. In this study, 80 mL of gelling solution were prepared and moved to a sample adapter that could be sealed and set it in an oven at a specific temperature. The apparent viscosity was measured at regular intervals by brookfield viscometer DVII. Figure 1 shows the crosslinking reaction process of the NPAM/PEI gel system. The gelation time was defined as the time corresponding to the inflection point on the plot of apparent viscosity versus time [10]. The gel strength was represented by the viscosity corresponding to the equilibrium period of the gelation curve.

#### 2.2.3. Statistical Analysis

The experiments were designed with two factors (polymer and crosslinker concentration) and five levels, as shown in Table 2. All experiments were carried out as a designed scheme, and the obtained data of gelation time were fed into the “datafit” software. A mathematical model was obtained by using this approach, which could be utilized to express the comprehensive relationship between the gelation time and NPAM and PEI concentration by the following second-order polynomial equation [28,29]:

(1) $$GT=\alpha_{0}+\overset{k}{\underset{i=1}{\sum }}\alpha_{i}C_{i}+\overset{k}{\underset{i,j=1}{\sum }}\alpha_{ii}C_{i}{}^{2}+\overset{k}{\underset{i,j=1}{\sum }}\alpha_{ij}C_{i}C_{j}+\varepsilon$$

where *GT* is gelation time; $C_{i}$ and $C_{j}$ are the NPAM and PEI concentrations, respectively; and $\alpha_{0}$, $\alpha_{i}$, $\alpha_{ii}$, $\alpha_{ij}$, and ε are intercept, linear, quadratic, and interaction constant coefficients, and the statistical random error term, respectively.

The correlation curve of experimental and predicted gelation time was plotted to assess the predictability of the generated model. Moreover, variance analysis (ANOVA) was performed to evaluate the significance of the developed model by Fish’s criterion (F-test), and the accuracy with a 95% confidence level was determined using *p*-value (probability error). The R-square and adjusted R-square were calculated to confirm the reliability of the model.

#### 2.2.4. Scanning Electron Microscope (SEM)

SEM was performed to observe the microstructure of NPAM/PEI gel. Before the test, the gel samples were dried and coated with gold palladium film in vacuum. Representative sections were photographed for evaluation.

#### 2.2.5. Thermal Gravimetric Analysis (TGA)

The thermal stability of NPAM/PEI gel system was measured by TGA in this study. The matured gel was dried and placed in hermetic pan after the gelling solution matured at 80 °C for three days. The measurement was conducted at the temperature range of 30–500 °C at the scanning rate of 5 °C/min.

#### 2.2.6. Core Flowing Experiment

**Figure 2:**
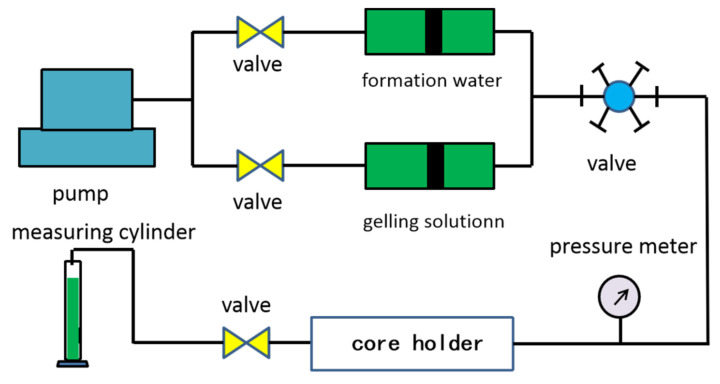
The schematic of core flowing experiment.

The core flowing experiments were conducted to determine the water control capacity of NPAM/PEI gel by plugging rate. The water flooding rate was set at 0.5 mL/min. The schematic of the core flowing experiment is shown in Figure 2, and the detailed experimental steps were as follows.

(1) The cores were washed, dried, and weighed. Injected water was used to saturate the cores in vacuum, and their pore volumes (PV) and porosity were determined using weight method.
(2) The water flooding was carried out until the inlet pressure and output fluid reached a stable condition. The initial permeability of the core was calculated according to Darcy’s law in the form:
  (2) $$k=\frac{q\mu L}{A\left(P_{1}-P_{2}\right)}$$
  where *k* is the permeability in mD, *q* is the flow rate in ml/min, μ is the viscosity of flooding water in mPa·s, *L* is the core length in cm, A is the cross-sectional area of core in cm^2^, and $P_{1},P_{2}$ are the pressures of the core inlet and outlet in MPa, respectively.
(3) The gelling solution (0.5 PV) was injected into the core, and water (0.5 PV) was injected to replace the gelling solution. After that, the core holder was sealed and placed at 80 °C until the gel matured.
(4) Regained-permeability testing was conducted by water flooding as Step (2). The plugging rate (ϕ) of NPAM/PEI gel system was evaluated using the following relation:
  (3) $$\phi =\frac{k_{0}-k_{1}}{k_{0}}\times 100\%$$

The residual resistance factor (RRF), which reflects the ability of plugging agent to reduce the permeability of porous medium, was computed as:

(4) $$\mathrm{RRF}=k_{0}/k_{1}$$

where $k_{0}$, $k_{1}$ denote the permeability of the core before and after gel treatment, respectively, in mD.

## 3. Results and Discussion

Two main factors, polymer and PEI concentration, were considered to evaluate the gelation performance of NPAM/PEI gel system. Each factor was varied in five levels, while other influencing parameters were kept constant. All gelling solutions were prepared in fresh water at room temperature, and the experiments were conducted at 80 °C. The specific experimental scheme and results are illustrated in Table 2. The low initial viscosity is observed as the NPAM concentration varies from 0.3 to 0.7 wt%, which gives good injectivity of the gelling solutions.

### 3.1. Mathematical Modeling of NPAM/PEI Gel System

Based on the data of gelation time (GT) in Table 2, the attempt was conducted on fitting the data of GT with a quadratic polynomial model (Equation (1)). A prediction model of gelation time was developed that is represented by the following equation:

(5) $$\mathrm{GT}=25.93-43.18C_{A}-316.42C_{B}+165.17C_{A}C_{B}+27.14C_{A}{}^{2}+1598.19C_{B}{}^{2}$$

In this model, the variables $C_{A}$ and $C_{B}$ represent polymer and crosslinker concentration, respectively. $C_{A}C_{B}$ is the interaction of the two variables. It can be seen that the interaction between the two variables have significant effects on the response (GT). In addition, the coefficients of the crosslinker terms in the model are higher than that of polymer terms, so that the effect of PEI on GT is greater than NPAM.

**Figure 3:**
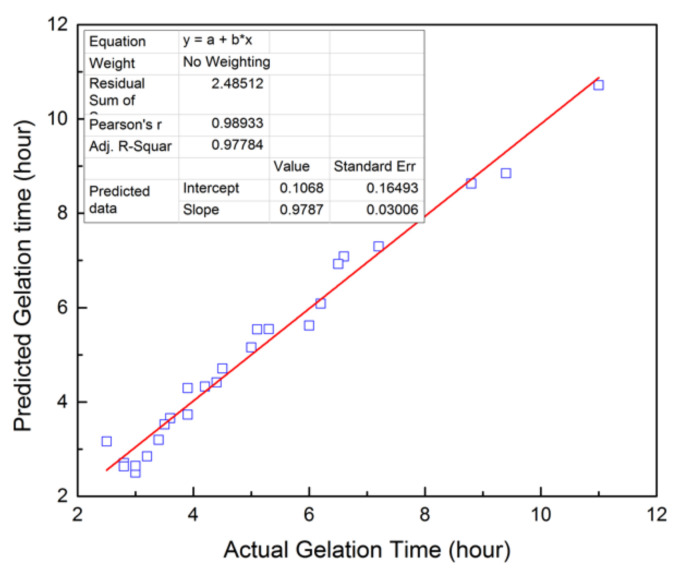
Predicted value based on the obtained model vs. actual value of GT.

The predicted and experimental gelation time is plotted in Figure 3. The slop of the linear fitting curve is 0.9787, very close to 1. This indicates that the predicted data are fitted well with the experimental data. The coefficients of multiple determination (R^2^) and adjusted multiple determination (R_a_^2^) for the developed model are 0.9788 and 0.9732 (Table 3), respectively, which verify the reliability of this model.

The statistical significance of the developed mathematical model was further assessed by ANOVA. As shown in Table 3, it can be concluded from the high F-ratio value (175.16) that the obtained model is highly significant. Furthermore, the *p*-value of <0.05 indicates the quadratic model is able to explain 95% of the variability in gelation time for the designed NPAM/PEI gel system.

### 3.2. Effects of Polymer and Crosslinker Concentration

**Figure 4:**
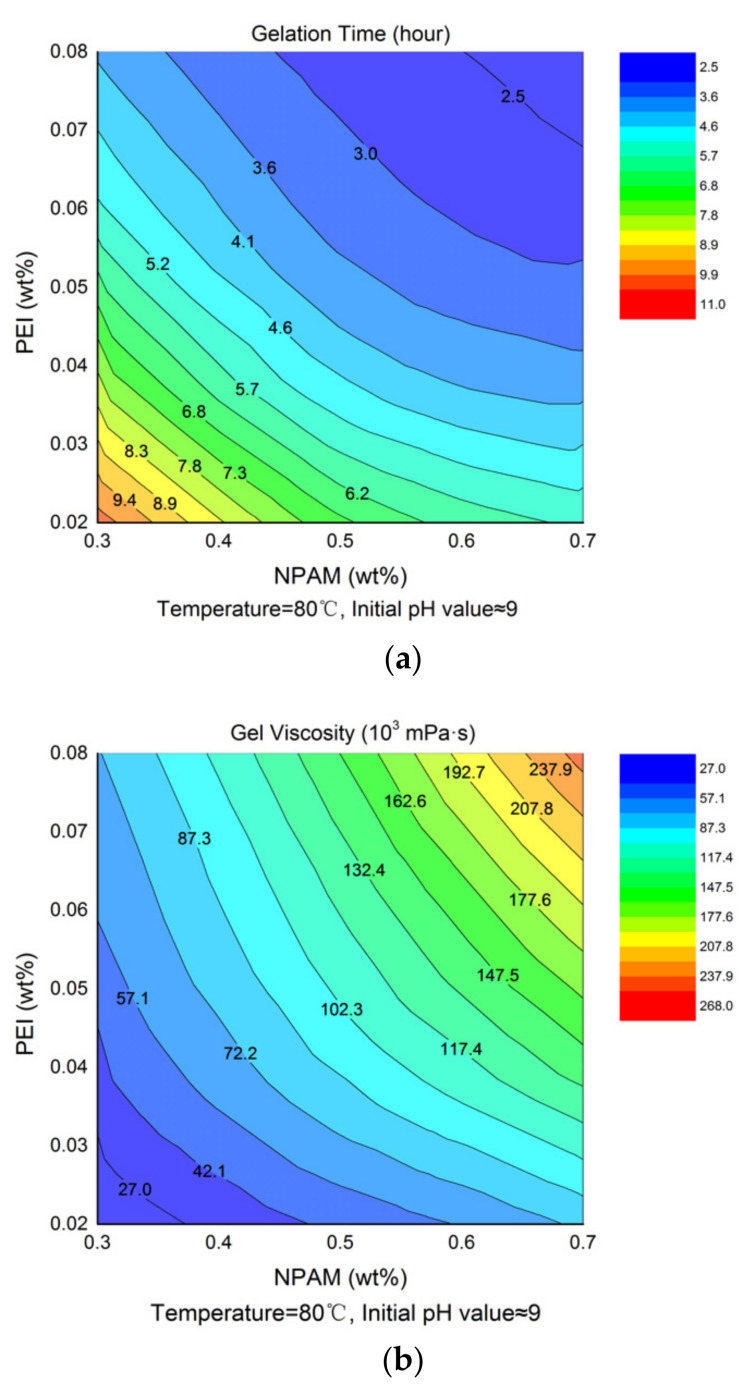
Contour map of gelation time and gel viscosity for NPAM/PEI gel system: (**a**) contour map of gelation time; and (**b**) contour map of gel viscosity.

The experimental data in Table 2 were extracted and plotted as a function of NPAM and PEI concentration, respectively. Figure 4a,b presents the contour maps of gelation time and gel viscosity, respectively, which reflect the interaction between polymer and crosslinker concentration. It is observed that the contour of gelation time varies from 11 to 2.5 h from bottom left to top right in Figure 4a, while the contour of gel viscosity correspondingly increases from 27.02 to 267.56 Pa·s in Figure 4b. The shorter gelation time and higher gel viscosity were obtained with the increment of NPAM concentration, as high polymer concentrations provide more crosslinking sites, which accelerate the crosslinking rate and make the crosslinking networks stronger. The increase of PEI concentration has a similar effect to that of the polymer on the gelation performance of NPAM/PEI gel system. That is because more amine groups are available for crosslinking with increasing PEI concentration. It should be noted that excessive PEI would cause the occurrence of over-crosslinking [19].

### 3.3. Effect of Retarder

**Figure 5:**
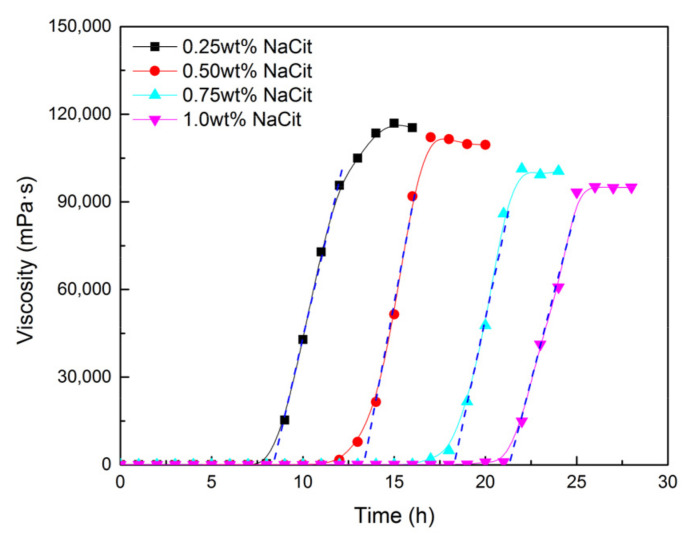
Effect of NaCit on gelation performance of NPAM/PEI gel system.

To prolong the gelation time of NPAM/PEI gel system and ensure it migrated into the depth of the reservoir, sodium citrate (NaCit) was added into the gelling solution as a retarder with the concentration ranging from 0.25 to 1.0 wt%. All samples were prepared with fresh water with the formulation of 0.5 wt% NPAM and 0.06 wt% PEI. The experiments were conducted at 80 °C, and the results are given in Figure 5. The gelation time of NPAM/PEI gel system prolonged significantly with the increment of NaCit concentration. Compared to the sample with salt-free (3.2 h), the gelation time was retarded to 21.3 h with the addition of 1.0 wt% NaCit, which increased by approximately 6.6 times. The gelation time varied linearly with the concentration of NaCit, and the relationship is expressed as follows:

(6) $$\mathrm{GT}=3.58+18.6C_{NaCit}$$

where $C_{NaCit}$ is the concentration of NaCit in mg/L.

**Figure 6:**
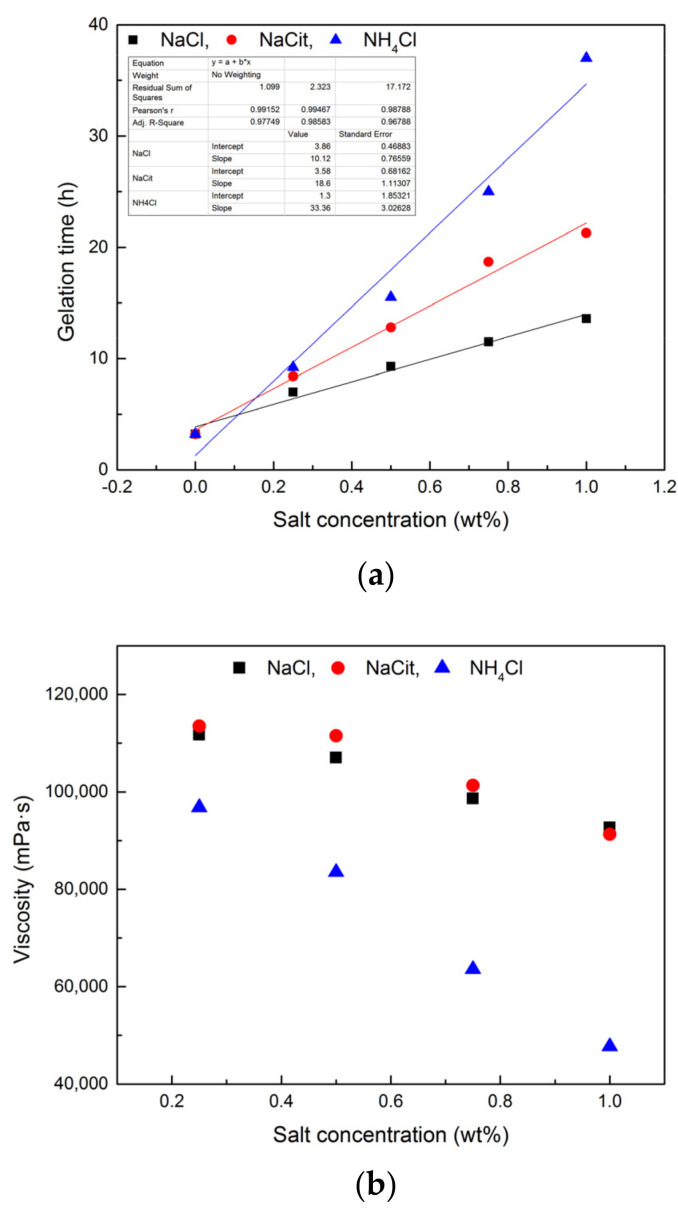
Comparison of different types of retarder: (**a**) the gelation time of NPAM/PEI gel with different retarders; and (**b**) the gel viscosity of NPAM/PEI gel with different retarders.

**Figure 7:**
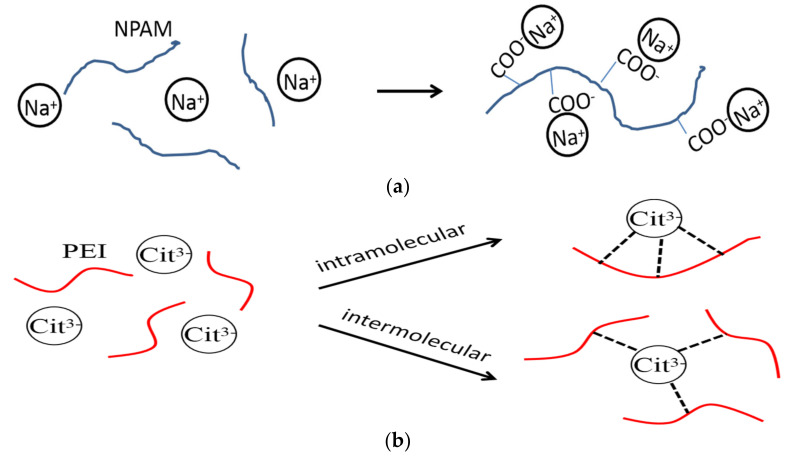
Schematic of interaction of sodium ions and citrate ions on NPAM/PEI gel system: (**a**) charge shielding effect of sodium ions on NPAM; and (**b**) complexation of citrate ions and PEI.

To further discuss the retarding capacity of NaCit on NPAM/PEI gel system, the samples in the presence of NaCit, NaCl, and NH_4_Cl were compared with the same mass fraction in Figure 6. It can be observed that the retarding capacity of NaCit is better than that of NaCl. Despite the addition of NH_4_Cl results in the longest gelation time, a significant reduction in gel viscosity is also observed, as shown in Figure 6b. The retarding effect of NaCl is mainly due to the charge shielding effect of Na^+^ on carboxyl groups (COO^−^) of NPAM chains, as shown in Figure 7a. However, the molar concentration of Na^+^ in NaCit is lower than that of NaCl at the same mass fraction. It suggests that the effect of NaCit on the gelation time of NPAM/PEI gel system is not only due to the charge screening of Na^+^, but the presence of citrate ions also plays a key role on the delaying of the gelation time. Avadiar reported the PEI molecular adsorbed on the surface of silica particles could remove the citrate ions in solution acted as a bridging agent [30]. It indicated that the interaction between citric ions and PEI due to the opposite charge of their molecules. Moreover, there are three carboxylate groups on the citrate ion. Two possible scenarios, intramolecular and intermolecular interaction, may occur between the carboxylate groups of citrate ions and the ammonium groups on PEI molecules, as illustrated in Figure 7b.

As reported in the literature, some delayed crosslinked gel systems have been developed. The HPAM/PEI gel in the presence of dextran sulfate (DS) could reach 120 h in gelation at 100 °C [24]. It is a promising retarder for PEI crosslinked polymer gel, but it is too expensive. In addition, the PECs which were developed from PEI and DS could elongate the gelation time of HPAM/Cr^3+^ gel system to seven days at 40 °C, while the gelation time was 6 h at 80 °C [31]. Compared with these retarders above, NaCit is low-cost and environmentally friendly.

### 3.4. Effect of Initial pH Value

**Figure 8:**
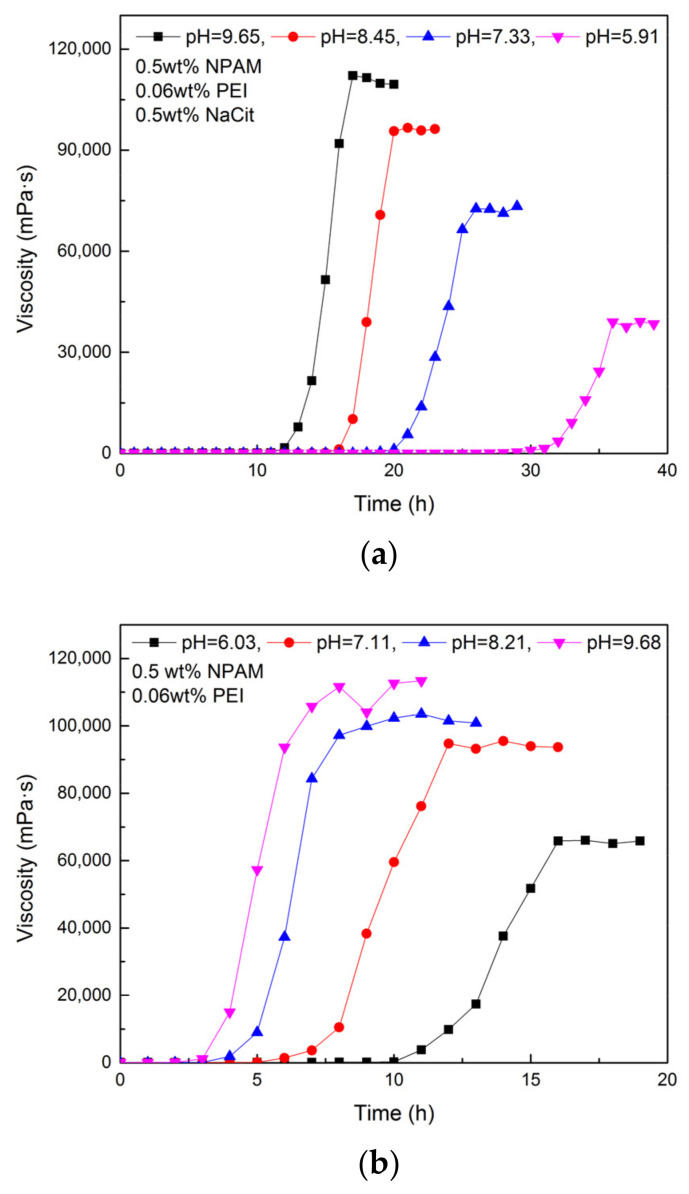
Gelation performance of NPAM/PEI gel system at different initial pH values: (**a**) the gel system with 0.5 wt% NaCit; and (**b**) the gel system without NaCit.

Two groups of gel system composed of 0.5 wt% NPAM and 0.06 wt% PEI were prepared with fresh water. To one of them was added 0.5 wt% NaCit, and the other was without NaCit. The pH value of these gelling solutions was adjusted by adding hydrochloric acid. The gelation performance of all samples was measured at 80 °C, and the results are shown in Figure 8. It can be seen that the gel system in acidic has a longer gelation time than that in alkaline and neutral. However, the gel viscosity tends to be weaker when the initial pH value varies from alkaline to acidic. This observation is due to the fact that many amine groups (-NH_2_) on the PEI chains are protonated in acidic and lost the activity of nucleophilic substitution, which leads to the decrease of the available crosslinking sites. Consequently, the longer gelation time and weaker gel viscosity are obtained. As reported by Suh et al., the unprotonated nitrogen in PEI chain in alkaline and neutral conditions was above 85%, while that in acidic decreased rapidly [32]. This result provides effective evidence for the above explanation. Compared with the gel system without NaCit, the gel system with 0.5 wt% NaCit is more sensitive to the variation of initial pH value. It may be because the interaction between citrate ions and PEI is promoted while the initial pH value of gelling solution decreases.

### 3.5. Effect of Temperature

Two groups of NPAM/PEI gel systems were prepared with the formulation of 0.5/0.06 wt%. To one group was added 0.5 wt% NaCit, and the other was without NaCit. The effect of temperature on the gelation process of the NPAM/PEI gel system was performed in the range of 50–80 °C. The experimental results are given in Table 4. As compared with the samples at 50 °C, the gelation time of the two formulas at 80 °C decreased by 5.5 and 5.6 times, respectively. It revealed that high temperature plays an accelerative effect on the gelation process of NPAM/PEI gel system. Moreover, increasing temperature is made to form a stronger gel. It is believed that the effective collision between polymer and PEI molecular enhances with increasing temperature, which improves the crosslinking rate and crosslinking density. Therefore, the higher gel viscosity and shorter gelation time are obtained.

The Arrhenius-type equation can be utilized to predict the gelation time with different temperature [16]:

(7) $$\mathrm{GT}=\mathrm{Aexp}\left(E/\mathrm{RT}\right)$$

where E is the activation energy in kJ/mol, R is the universal gas constant in kJ/(mol·K), T represents the absolute temperature in K, and A is the frequency factor in hour. Logarithmic transformation of Equation (7) can be expressed as:

(8) $$\ln\left(\mathrm{GT}\right)=\mathrm{lnA}+E/\mathrm{RT}$$

**Figure 9:**
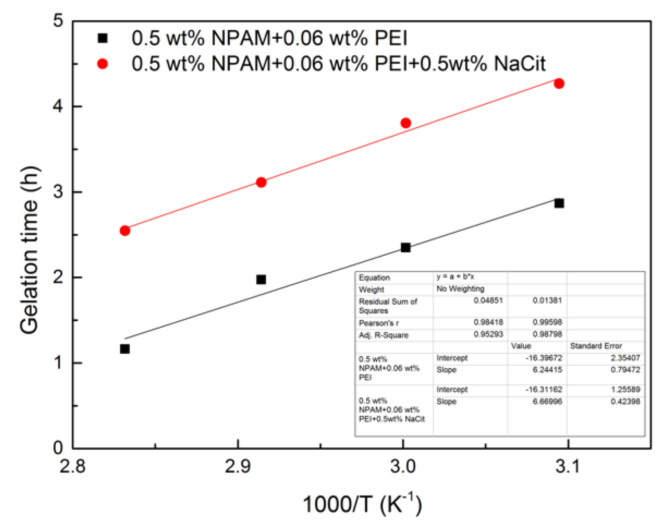
The plot of ln(GT) vs. 1000/T.

The logarithm data of GT in this study is extracted and fitted to the reciprocal of absolute temperature, and the plot of ln(GT) vs. 1/T is given in Figure 9. The data of GT are fitted well with Equation (8). The activation energies of two formulas were calculated to be 51.91 and 55.45 kJ/mol, respectively. It indicates that the addition of NaCit has little influence on the activation energy of the crosslinking reaction between NPAM and PEI. These values are lower than the data obtained by Al-Muntasheri et al. [16], who found that NPAM with high molecular weight is easier to react with PEI.

### 3.6. Compatibility with the Injection and Formation Water

**Figure 10:**
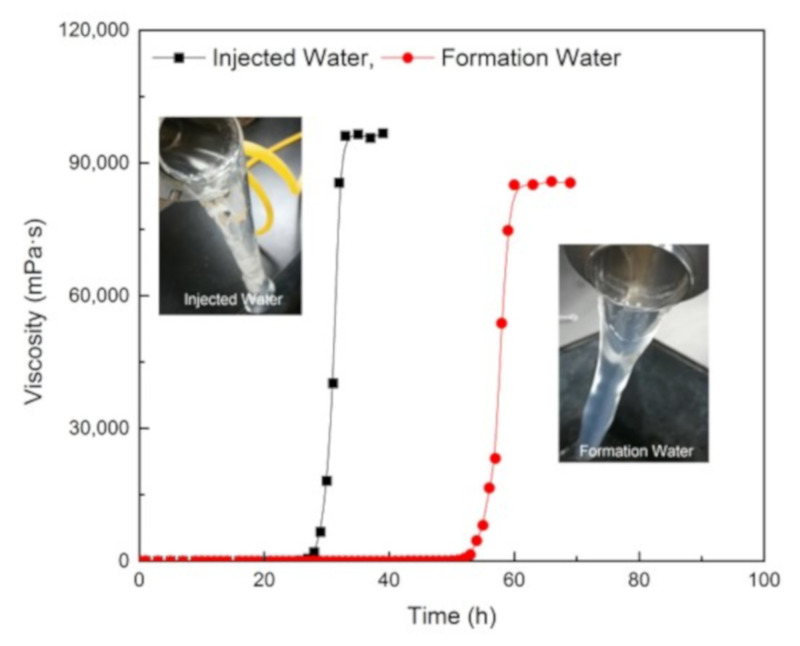
Gelation performance of NPAM/PEI/CitNa gel system in injection and formation water.

Two gelling solutions were prepared with the injection and formation water, respectively. The gel system was composed of 0.5 wt% NPAM, 0.06 wt% PEI, and 0.5 wt% NaCit. The gelation process of these two samples was measured at 80 °C, and the results are shown in Figure 10. Comparing with the gel prepared with fresh water (Figure 5, 0.5 wt% NaCit), two gels in these experiments have a longer gelation time, and the equilibrium viscosity of the gel prepared with formation water decreases significantly. That is because a large amount of metal cations in the formation water plays a strong charge shielding effect on NPAM molecules, causing the polymer molecular chains to coil seriously, which reduced the available crosslinking amide groups (-CONH_2_). Nevertheless, both gels are observed to form the bulk gels and show good viscoelasticity property as suggested by the tongue phenomenon. It indicates that the NPAM/PEI/NaCit gel system has a good compatibility with the injection and formation water.

### 3.7. Microstructure of the Bulk Gel

**Figure 11:**
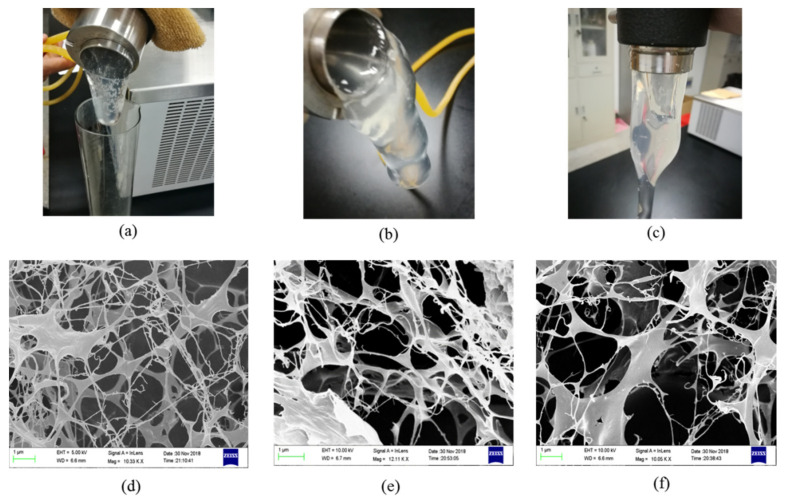
Microstructures of the gel systems with different formulations: (**a**,**d**) Sample 1, the gel composed of 0.7 wt% NPAM, 0.08 wt% PEI, and 0.5 wt% NaCit; (**b**,**e**) Sample 2, the gel composed of 0.5 wt% NPAM, 0.06 wt% PEI, and 0.5 wt% NaCit; and (**c**,**f**) Sample 3, the gel composed of 0.5 wt% NPAM, 0.06 wt% PEI, and 1.0 wt% NaCit.

Three gel samples were prepared with different formulations at 80 °C, and their microstructures were observed by SEM. Sample 1 was the gel composed of 0.7 wt% NPAM, 0.08 wt% PEI, and 0.5 wt% NaCit. Sample 2 was the gel prepared with 0.5 wt% NPAM, 0.06 wt% PEI, and 0.5 wt% NaCit. Sample 3 was the gel consisted of 0.5 wt% NPAM, 0.06 wt% PEI, and 1.0 wt% NaCit. As shown in Figure 11a–c, the bulk gels are formed in three samples. The remarkable tongue phenomenon of crosslinked polymer gel is observed in all three samples while gradually inverting the sample adapter containing the gel sample. It indicates that the formed gels possess good viscoelasticity, and the gel with high NPAM and PEI concentration is more difficult to flow due to its high gel viscosity. Figure 11d–f shows the microstructures of these three gel samples. It can be seen that the gel composed of high NPAM and PEI concentration has denser microstructure. The microscopic pore size of Sample 1 is mostly around 1 μm while that of Sample 2 is distributed at 1–3 μm. Comparing with Sample 2, the network pore size in Sample 3 is enlarged to 2–4 μm. It indicates that increasing NaCit concentration results in the decrease of the crosslinking density of the NPAM/PEI gel system. This result is consistent with the gel viscosity shown in Figure 5.

In all three samples with different formulations, the dense three-dimensional network is built by the transamidation of amide groups of NPAM and amine nitrogen of PEI. This kind of microstructure is conductive to lock the free water and keep the gel stable. When this microstructure is formed in formation, it contributes to bridge the pore throats of the formation and restricts the flow of water in high permeability zone of the formation.

### 3.8. Thermal Stability of NPAM/PEI Gel System

**Figure 12:**
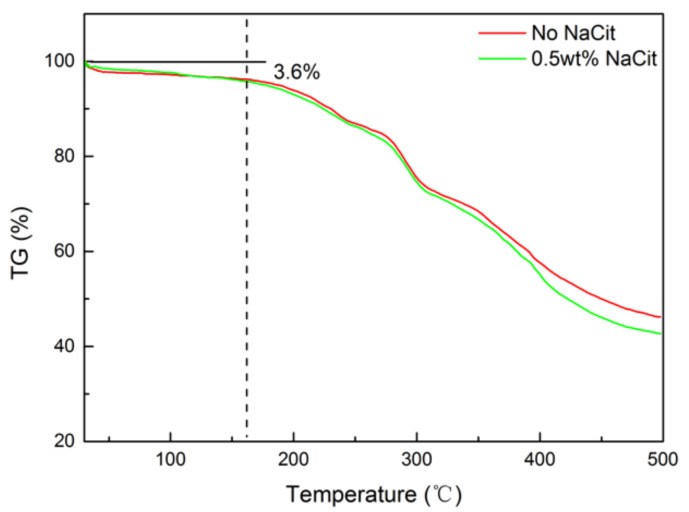
TGA curves of the NPAM/PEI gels.

Two matured gels were composed of 0.5 wt% NPAM and 0.06 wt% PEI. One was prepared without NaCit, and to the other was added 0.5 wt% NaCit. TGA were conducted to evaluation the thermal stability of these two samples. Figure 12 shows the TGA curves of the two NPAM/PEI gel. It can be seen that the TGA curves of the two samples almost coincide below 300 °C. The mass loss rate of the gel with 0.5 wt% NaCit slightly enhances at the temperature above 300 °C. It indicates that the presence of NaCit in this gel system has little influence on the thermal stability. The mass loss of the two dried gels are approximately 3.6% as the temperature increases from 30 to 160 °C. It may be due to the evaporation of residual bound water in dry gel powder. This observation indicates that the gel system can keep stable below 160 °C. When the temperature exceeds 160 °C, the mass loss accelerates significantly. It suggests that the gel component starts to decompose. Therefore, the gel system should be applied below 160 °C.

### 3.9. Plugging Capacity of NPAM/PEI Gel System

Two cores with similar permeability were selected and numbered Core 1 and Core 2, and the plugging capacity of two gel systems was evaluated by the core flowing experiment. The injected water was used as the displacement water in these experiments. As shown in Table 5, the variation of the permeability before and after gel injection suggests that the gel system effectively blocks the high permeability channels of the cores after matured and drives the subsequent brine water into the low permeability zone. Consequently, the high water flow resistance is obtained. The plugging rates of the two gels are more than 95%, and their residual resistance factors (RRF) are above 25. The results indicate that NPAM/PEI gel system has a good plugging capacity. Moreover, the higher is the gel viscosity, the larger is the plugging rate. The plugging mechanism of crosslinked polymer gel mainly depends on the retention of gel in high permeability channel and adsorption on the rock surface [18]. The higher residual resistance factor suggested that the stronger gel generally has better blocking effect in porous media.

## 4. Conclusions

The gel system composed of NPAM, PEI, and NaCit shows low initial viscosity (<75 mPa·s), adjusted gelation time (2.5–21 h), and gel viscosity (27.02–267.56 × 10^3^ mPa·s). A prediction model of gelation time which reflects the effect of NPAM and PEI and their interaction was developed through the statistical analysis. The mathematical model fit perfectly with the experimental data. The obtained model is highly significant, as approved by the F-ratio of 175.16, and is capable of explaining 95% of the variability in gelation time (*p*-value of <0.05). The reliability and accuracy of the model are verified by the high R-square value (0.9788) and high adjusted R-square value (0.9732). The addition of NaCit effectively retards the gelation time of NPAM/PEI gel system, which is due to the synergy of the charge shielding effect of sodium ions on NPAM molecules and the complexation of citrate ions and PEI. The gelation time of this gel system is elongated as the initial pH value varies from alkaline to acid, whereas the gel viscosity tends to be weaker. Increasing temperature accelerates the gelation process of the NPAM/PEI gel system. The dense microstructures of the matured gels are observed, and they can keep stable below 160 °C. Moreover, the NPAM/PEI gel system shows good compatibility with the injection and formation water. The permeability of the core is reduced more than 95% and the water flow resistance increases significantly after the NPAM/PEI gel formed in the natural cores. Thus, the delayed crosslinked polymer gel composed of NPAM, PEI, and NaCit has potential application for in-depth water control in mature oilfields.

## Figures and Tables

**Table 1:** The ionic content of injected and formation water.

Water Type	CO_3_^2−^ (mg/L)	HCO_3_^−^ (mg/L)	Cl^−^ (mg/L)	SO_4_^2−^ (mg/L)	Ca^2+^ (mg/L)	Mg^2+^ (mg/L)	Na^+^/K^+^ (mg/L)	TDS (mg/L)
Injected water	1216.25	—	274.38	27.03	95.65	15.27	984.6	2613.18
Formation water	6792.4	146.7	52.44	71.69	43.36	27.64	10,468.7	17,602.93

**Table 2:** Experimental scheme and results.

NPAM Concentration (wt%)	PEI Concentration (wt%)	Initial Viscosity (mPa·s)	Gelation Time (hour)	Gel Viscosity (×10^3^ mPa·s)
0.3	0.02	11.8	11	27.02
	0.03	11.4	9.4	33.726
	0.04	11.4	7.2	40.081
	0.06	10.7	5	69.547
	0.08	10.5	3.9	55.242
0.4	0.02	16.0	8.8	32.254
	0.03	15.8	6.5	38.274
	0.04	15.3	5.1	70.508
	0.06	16.5	3.9	80.832
	0.08	14.8	3.4	102.587
0.5	0.02	22.3	6.6	46.676
	0.03	22.5	5.3	70.641
	0.04	23.4	4.2	102.708
	0.06	25.2	3.2	111.609
	0.08	22.6	3	137.169
0.6	0.02	46.9	6.2	49.249
	0.03	43.2	4.5	87.003
	0.04	43.9	3.6	123.668
	0.06	45.4	3	135.19
	0.08	44.8	2.8	203.426
0.7	0.02	73.0	6	53.054
	0.03	73.1	4.4	105.372
	0.04	72.7	3.5	160.43
	0.06	74.3	2.8	182.256
	0.08	71.4	2.5	267.564

**Table 3:** The results of ANOVA.

ANOVA	Sum of Square	DOF	Mean Square	F-Ratio	*p*-Value
Regression	117.04	5	23.41	175.16	<0.0001
Residual	2.54	19	0.1336		
Total	119.58	24			
Data fit	R^2^	R_a_^2^	Residual error		
	0.9788	0.9732	0.3656		

**Table 4:** Experimental results at different temperatures.

Gel Systems	Temperature (°C)	Gelation Time (hour)	Viscosity (×10^3^ mPa·s)	R^2^	E_a_ (kJ/mol)
Formula 1: 0.5 wt% NPAM + 0.06 wt% PEI	50	17.6	57.686	0.9529	51.91
	60	10.5	74.852		
	70	7.2	104.703		
	80	3.2	111.609		
Formula 2: 0.5 wt% NPAM + 0.06 wt% PEI + 0.5 wt% NaCit	50	71.5	53.62	0.9880	55.45
	60	45	81.86		
	70	22.5	101.435		
	80	12.8	109.523		

**Table 5:** The plugging capacity of NPAM/PEI gel system.

Gel System	Core	Size		Porosity %	Permeability (×10^−3^ μm^2^)		Plugging Rate (%)	RRF
		Length (cm)	Diameter (cm)		Before Gel Injection	After Gel Injection		
0.5 wt% NPAM + 0.06 wt% PEI + 0.5% NaCit	Core 1	4.74	2.54	28.73	66.88	2.35	96.49	28.45
0.7 wt% NPAM + 0.08 wt% PEI + 0.5% NaCit	Core 2	4.88	2.54	28.50	68.82	1.58	97.70	43.56
